# ICSBP-induced PD-L1 enhances osteosarcoma cell growth

**DOI:** 10.3389/fonc.2022.918216

**Published:** 2022-09-23

**Authors:** Jee Young Sung, June Hyuk Kim, Hyun Guy Kang, Jong Woong Park, Seog-Yun Park, Byung-Kiu Park, Yong-Nyun Kim

**Affiliations:** ^1^ Metastasis Branch, Division of Cancer Biology, National Cancer Center, Goyang, South Korea; ^2^ Orthopedic Oncology Clinic, Center for Rare Cancers, National Cancer Center, Goyang, South Korea; ^3^ Pathology Department, National Cancer Center, Goyang, South Korea; ^4^ Center for Pediatric Oncology, National Cancer Center, Goyang, South Korea

**Keywords:** osteosarcoma, programmed death-ligand 1 (PD-L1), interferon consensus sequence binding protein (ICSBP), cell cycle, apoptosis

## Abstract

**Background:**

Interferon (IFN) consensus sequence binding protein (ICSBP) is a transcription factor induced by IFN-γ. We previously reported that ICSBP expression promotes osteosarcoma progression by enhancing transforming growth factor-β signaling. In cancer cells, programmed death-ligand 1 (PD-L1) contributes to immune escape and may also be involved in tumor progression. Because IFN-γ induces the expression of both ICSBP and PD-L1, we explored the association between ICSBP and PD-L1 expression in terms of osteosarcoma progression.

**Methods:**

Three osteosarcoma cell lines (Saos2, U2OS, and 143B) were employed. Gene expression was measured by qRT-PCR, and protein levels were assessed by immunoblotting. PD-L1 expression was evaluated in cells overexpressing ICSBP and in ICSBP knockdown cells. The effects of PD-L1 expression on cell growth were examined by MTS assays, Incucyte analysis, soft agar assays, and three-dimensional (3D) culture. Cell cycle and apoptosis were evaluated by FACS analysis of cells stained with propidium iodide (PI) and annexin V/PI, respectively. The antitumor effects of PD-L1 knockdown without or with doxorubicin treatment were evaluated *in vivo* in nude mice bearing ICSBP-overexpressing 143B cell xenograft. The clinical relevance of PD-L1 and ICSBP expression was evaluated immunohistochemically using a human osteosarcoma microarray and through analysis of publicly available data using Gene Expression Profiling Interactive Analysis2.

**Results:**

ICSBP overexpression upregulated PD-L1 expression in all three cell lines, whereas ICSBP knockdown decreased the PD-L1 expression. PD-L1 knockdown attenuated the cell growth and reduced colony-forming capacity in both soft agar assays and 3D culture. PD-L1 knockdown increased apoptosis and induced G2/M arrest, which was associated with decreased expression of survivin, cyclin-dependent kinase 4 (CDK4), cyclin E, and cyclin D1 expression and increased the expression of p27, phosphorylated Cdc2, and phosphorylated Wee1. PD-L1 knockdown decreased the growth of tumor xenografts and increased the doxorubicin sensitivity of ICSBP-overexpressing 143B cells both *in vitro* and *in vivo.* PD-L1 was expressed in human osteosarcoma tissues, and its expression was moderately correlated with that of ICSBP in osteosarcoma patients.

**Conclusion:**

ICSBP regulates PD-L1 expression in osteosarcoma cells, and PD-L1 knockdown combined with doxorubicin treatment could represent a strategy for controlling osteosarcoma expressing ICSBP.

## Introduction

Osteosarcoma is the most common pediatric bone malignancy (1) and is a highly metastatic disease (2). Despite great advances in tumor therapeutics, the osteosarcoma survival rate has remained stagnant for the last several decades. Furthermore, recurrent or metastatic osteosarcomas are mostly resistant to chemotherapy and their outcome is dismal (3). Therefore, a new therapeutic modality is urgently needed. Immune checkpoint inhibitors, such as those targeting cytotoxic T lymphocyte-associated-4 (CTLA-4) and programmed cell death protein 1 (PD-1)/programmed death-ligand 1 (PD-L1), have recently been developed (4) and tested, both alone and in combination with other targeted therapy, chemotherapy, or radiotherapy in various malignancies, including lung cancer, melanoma, head and neck cancer, genitourinary cancer, and lymphoma (5). Immune checkpoint inhibitors have also been assessed against osteosarcoma in a few clinical trials but with disappointing results (6). One clinical trial examining the efficacy of the PD-1 inhibitor pembrolizumab in recurrent osteosarcoma patients reported a response rate of only 5% (6). Other trials examining nivolumab (PD-1 inhibitor) plus ipilimumab (CTLA-4 inhibitor) and pembrolizumab plus cyclophosphamide (Immune system suppressor) for osteosarcoma therapy are ongoing (7).

PD-L1 is induced by interferon-gamma (IFN-γ), which is secreted by T cells and natural killer cells (8). IFN-γ activates signal transducer and activator of transcription (STAT)1/3 molecules through the Janus kinase (JAK)1/2 pathway. The activation of STAT proteins enhances the binding of IFN regulatory factor 1 (IRF1) to the PD-L1 promoter, triggering PD-L1 expression (9). PD-L1 on the tumor surface binds PD-1 on cytotoxic T lymphocytes (CTLs), inactivating them (10) and allowing tumor cells to escape the host immune attack (10). Multiple lines of evidence indicate that PD-L1 expression is associated with tumor progression. PD-L1 expression is associated with poor prognosis in lung cancer (11). PD-L1 is overexpressed in triple-negative breast cancer (12) and is associated with osteosarcoma drug resistance and metastasis (13). PD-L1 is also expressed in metastatic osteosarcoma tissues, suggesting that it might have a role in metastasis, and a recent meta-analysis of solid tumor patients revealed an association between PD-L1 expression and reduced survival (14).

Despite evidence that PD-L1 contributes to osteosarcoma growth and progression, clinical trials using checkpoint inhibitors have been associated with disappointing outcomes, raising doubts regarding the efficacy of targeting PD-L1 (15). Because multidrug chemotherapy increases the disease-free survival of osteosarcoma patients, we tested the apoptotic effects of the combination of chemotherapeutic agents against osteosarcoma in the present study.

IFN consensus sequence binding protein (ICSBP), also known as IRF8, is a transcription factor in the IRF family induced by IFN-γ (16). ICSBP binds the IFN-stimulated response element to regulate gene expression during myeloid and B-cell differentiation (17). ICSBP also plays an essential role in macrophage maturation (18). We previously reported that ICSBP enhances tumorigenicity and tumor progression in human osteosarcoma cells (19, 20). ICSBP forms heterodimers with other transcription factors, allowing it to function as either a transcriptional activator or a repressor, based on its interacting partners. For example, the interaction between ICSBP and IRF1 mediates the activation of genes involved in macrophage maturation (21). Because IRF1 is a well-known transcriptional activator of PD-L1, and both ICSBP and PD-L1 are induced by IFN-γ, ICSBP may also be involved in the regulation of PD-L1 expression. We examined whether ICSBP-induced PD-L1 mediates the observed effects of ICSBP in promoting the tumorigenicity and tumor progression of human osteosarcoma cells.

In the present study, we demonstrate that PD-L1 induced by ICSBP promotes osteosarcoma growth and tumorigenicity and that PD-L1 knockdown combined with doxorubicin treatment synergistically inhibits osteosarcoma growth, both *in vitro* and *in vivo*.

## Materials and methods

### Cell lines and culture

Human osteosarcoma cell lines MG63, HOS, 143B, U2OS, and Saos2 were obtained from the American Type Culture Collection (Rockville, MD, USA). Cells were maintained in RPMI 1640 medium (Life Technologies, Grand Island, NY, USA) supplemented with 10% fetal bovine serum (FBS; Life Technologies), 100 units/ml penicillin, and 100 μg/ml streptomycin (Life Technologies) at 37°C in a humidified incubator with an atmosphere containing 5% CO_2_.

### Establishment of ICSBP-expressing stable cell lines

The ICSBP PCR product was cloned into the *Hin*dIII and *Xho*I sites of the pcDNA3.1/V5-HisA vector (Invitrogen, MA, USA). 143B and U2OS cells were transfected with the ICSBP construct or empty vector (Mock) using Lipofectamine 2000 (Life Technologies) according to the manufacturer’s instructions. Stable cell lines, including 143B-Mock and 143B-ICSBP cells, were established by selection with 500 μg/ml of geneticin (G418, Calbiochem, La Jolla, CA, USA) for 4 weeks.

### Antibodies and reagents

Polyclonal antibodies against PD-L1, Ki67, PARP, caspase3, survivin, Bax, CDK4, Cyclin D1, Cyclin E, Cleaved caspase 3, GAPDH, and β-actin were purchased from Cell Signaling Technology (Beverly, MA, USA). The ICSBP antibodies (anti-goat and anti-mouse) were obtained from Santa Cruz Biotechnology (Santa Cruz, CA, USA). The PD-L1-PE antibody was purchased from eBioscience (Thermo Fisher Scientific, Waltham, MA, USA), and the polyclonal antibody against vinculin was purchased from Abcam (Cambridge, UK). Doxorubicin was purchased from Sigma (St. Louis, MO, USA).

### Immunoblot analyses

After washing with ice-cold PBS (10 mM Na_2_HPO_4_ pH 7.4, 145 mM NaCl, and 2.7 mM KCl), cells were lysed with 2× sodium dodecyl sulfate (SDS)-PAGE sample buffer (20 mM Tris pH 8.0, 2% SDS, 2 mM DTT, 1 mM Na_3_VO_4_, 2 mM EDTA, and 20% glycerol) and boiled for 5 min. The protein concentration of each sample was determined by using the Micro-Bicinchoninic Acid Protein Assay Reagent as described by the manufacturer (Thermo Scientific, Rockford, IL, USA). Total cellular protein (30 μg/lane) was separated by 10% SDS-PAGE and transferred to polyvinylidene difluoride (PVDF) membranes. The membranes were blocked overnight at 4°C in TBST (20 mM Tris pH 8.0, 150 mM NaCl, and 0.05% Tween 20) containing 5% non-fat milk. Membranes were then incubated overnight at 4°C with a primary antibody, washed three times with TBST, incubated with horseradish peroxidase (HRP)-conjugated goat anti-rabbit IgG secondary antibody for 1 h at room temperature, and washed three times with TBST. Proteins were visualized using an enhanced chemiluminescence reagent (Millipore).

### RNA interference

siRNAs specific for human ICSBP (si-ICSBP) and non-specific siRNA (si-Cont) were purchased from Dharmacon (Lafayette, CO, USA). siRNAs specific for human PD-L1(si-PD-L1) were purchased from Bioneer (Daejeon, Korea). Cells were transfected with 10 nM si-RNA using Lipofectamine RNAiMAX Reagent (Thermo Scientific, Waltham, MA) according to the manufacturer’s guideline. After 24 h of transfection, knockdown efficiency and specificity of each si-RNA were confirmed by using immunoblotting with corresponding antibodies.

### Establishment of sh-PD-L1 cell lines

PD-L1 shRNA lentiviral transduction particles (TRCN0000002746 and TRCN0000002749) and non-targeting shRNA lentiviral transduction particles (pLKO.1-puro Non-Target Control [SHC016V]) and non-targeting shRNA lentiviral transduction particles (pLKO.1-puro Non-Target Control [SHC016V]) were obtained from OriGene. Lentiviral particles were added in cultured cells. After 48 h, media were changed to fresh media with 2 μg/ml puromycin. The media were replaced every third day with fresh puromycin-containing media until stable clones were identified. PD-L1 knockdown was confirmed using immunoblotting analysis.

### Establishment of xenograft mice with either siRNA

143B-ICSBP cells (1 × 10^6^) were transfected with control siRNA(si-Cont) or specific PD-L1 si-RNA (si-PD-L1) for 24 h and then injected subcutaneously into male Balb/c nude mice. The greatest longitudinal diameter (length) and the greatest transverse diameter (width) of each tumor were determined once a week using a caliper. Each tumor volume was calculated by the modified ellipsoidal formula (tumor volume = tumor length × width × height × 0.52). We monitored tumor size twice in a week for up to 4 weeks. When the average tumor volume reached 30 mm^3^, tumor-bearing mice received intraperitoneal injection with either PBS or 5 mg/kg of doxorubicin twice a week for 4 weeks. This study was reviewed and approved by the Institutional Animal Care and Use Committee (IACUC) of National Cancer Center Research Institute (NCCRI).

### Immunohistochemical staining

Tumor tissues were fixed with 10% neutral buffered formalin. Formaldehyde-fixed specimens were paraffin-embedded and cut to a thickness of 4 μm. Sections were dried at 56°C for 1 h, and immunohistochemical staining was performed with Discovery XT (Ventana Medical Systems, Tucson, Arizona, USA) as follows. Sections were deparaffinized, rehydrated with EZ Prep (Ventana Medical Systems), and washed with reaction buffer. Antigens were retrieved with heat treatment in Tris-ethylenediaminetetraacetic acid (EDTA) pH 8.0 buffer (CC1, Ventana Medical Systems) at 90°C for 30 min for specific antibodies, anti-PD-L1 (1:100 dilution; Cell Signaling Technology), and anti-Ki67 antibodies (1:200 dilution; Cell Signaling Technology). Parallel sections incubated with normal IgG instead of primary antibodies were used as negative controls. The overall staining results were scored from 0 to 3 based on the intensity and positive rate of staining. Intensity of staining was categorized as 0, negative (-); 1, weak (_+); 2, intermediate (++); 3, strong (+++). Stained tissue arrays were reviewed by two experienced pathologists.

### Quantitative real-time PCR

Total RNA was extracted from the samples with Ribospin (GeneAll, Seoul, Korea). One microgram of total RNA was converted into cDNA by using HelixCript Easy cDNA Synthesis Kit (Nanohelix), and quantitative PCR analysis was performed on the Roche Light Cycler^®^ 96 by the SYBR Green qPCR method (Nanohelix). All reactions were performed in triplicate, and the relative transcript abundance of each tested gene was normalized to the expression level of housekeeping genes. cDNA fragments were amplified using the following primer pairs: human ICSBP, 5′-GGATATGCCTATGACACA-3′ (sense) and 5′-CATCCGGCCCATACAACTTAG-3′ (anti-sense); human PD-L1, 5′-ATCACTATCCCATTAGACACATC-3′ (sense) and 5′-CAAGAAACAGTTGACTTACGATT-3′ (anti-sense); and human GAPDH, 5′-ACTCAACACGGGAAACCTCA-3′ (sense) and 5′-AACCAGACAAATCGCTCCAC-3′ (anti-sense). Relative expression was calculated by the comparative Ct method. [2(-ddCt)] of each molecule was calculated as follows: dCt = Ct (molecule) – Ct (GAPDH); ddCt = dCt (experiment or target) – dCt (control or reference).

### Cell proliferation assay

For the cell proliferation assay, cells were transfected with si-control and si-PD-L1 and then plated at 3 × 10^3^/well in 96 wells, and cell growth was determined using Incucyte™ for 72 h and the CellTiter 96 Kit (MTS, 3-(4,5-dimethylthiazol-2-yl)-5-(3-carboxyme-thoxyphenyl)-2-(4-sulfophenyl)-2H-tetrazolium; Promega, Madison, WI, USA), as previously described in the manufacturer’s instruction. Afterward, 20 μl of the MTS solution was treated to each well, and the plates were incubated for another 2 h. The absorbance was measured at 490 nm with a PowerWave HT spectrophotometer (Biotek Instruments, Winooski, VT). Each experiment was conducted in triplicate.

### Colony-forming assay

Cells were transfected with si-control and si-PD-L1 for 24 h, and cells (4 × 10^2^) were plated in six wells in 2 ml fresh RPMI medium containing 10% FBS. After 7 days, colonies were fixed with 4% formaldehyde and stained with crystal violet solution and images were taken using a light microscope (Olympus, Narishige, Japan) and the number of colonies (>0.5 mm) were counted using ImageJ software (Version 1.8.0) from three independent experiments.

### Flow cytometry analysis

Cells were trypsinized and suspended in PBS containing 2.5 mM EDTA, 2.5 mM EGTA, and 1% BSA. For the apoptosis assay, treated cells were harvested and incubated for 15 min at RT with FITC-conjugated annexin-V reagent (2.5 μg/ml) and propidium iodide (PI) (5 μg/ml) in binding buffer followed by flow cytometer analysis. Apoptotic cell death was analyzed by the total percentage of early and late apoptotic cells in different groups. For the cell-cycle analysis, cells were fixed with 70% ethanol for 2 h at 4°C and stained with PI solution. Cell-cycle distributions were analysis with PI contents in the cells with flow cytometer analysis. The data were analyzed with CellQuest Software (BD Biosciences, San Jose, CA). Data are means ± SD of three independent experiments.

### Wound healing

Cells transfected with si-control and si-PD-L1 for 24 h were seeded in six-well plates and cultured to 80%–90% confluence. Cells were pretreated with either control (PBS) or doxorubicin for 1 h. The cellular layer in each plate was scratched using a plastic pipette tip. After re-treatment, the migration of the cells at the edge of the scratch was analyzed at 12 h. The images of the cells were captured using phase-contrast microscopy.

### 3D culture assay

Cells were transfected with si-control and si-PD-L1 for 24 h. 3D culture assays were performed in eight-well chamber slides (Nunc™ Lab-Tek™, Thermo Fisher Scientific, Waltham, MA) by placing 2 × 10^3^ cells in 300 μl of 5% Matrigel onto a base layer 100 μl of 100% Matrigel. The plates were then incubated in a 5% CO_2_ atmosphere at 37°C for 8 days. Images were taken by using an Axio Observer Z1 fluorescence microscope (Carl Zeiss Microimaging, Thornwood, NY) and an Axion Vision camera (Axion Technologies, Houston, TX).

### Immunohistochemical staining for osteosarcoma tissue microarray

Osteosarcoma tissue arrays were obtained from SuperBioChips Laboratories (Seoul, Korea), which has been described previously (22). Each array contained 60 sections obtained from 60 patients by biopsy or surgical resection. The primary antibodies used were polyclonal rabbit PD-L1 (1:100 dilution; Cell Signaling Technology). The tumor tissues were incubated with primary antibodies for 1 h and then treated with an anti-rabbit biotinylated antibody (1:1,000 dilutions; Vector Laboratories) for 1 h. Color reaction was developed by incubation with diaminobenzidine solution (Sigma, St. Louis, MO), followed by counter staining with hematoxylin. Parallel sections incubated with normal IgG instead of primary antibodies were used as negative controls. The overall staining results were scored from 0 to 3 based on the intensity and positive rate of staining. Intensity of staining was categorized as 0, negative (-); 1, weak (+); 2, intermediate (++); 3, strong (+++). Stained tissue arrays were reviewed by experienced pathologists.

### Clinical data analysis

Clinical data and RNA-seq data from TCGA project were analyzed by GEPIA2 (23). Pairwise gene expression correlation was analyzed for given sets of TCGA sarcoma expression data, using Spearman.

### Statistical analysis

Comparison between two groups were performed using Student’s t-test. Significance was calculated by two-way ANOVA analysis followed by Bonferroni’s *post-hoc* test. Statistical significance was defined as *P < 0.05 and **P < 0.01. Data represent average values and standard deviations (error bars) obtained from three independent experiments.

## Results

### PD-L1 is induced by ICSBP in osteosarcoma cells

**Figure 1:**
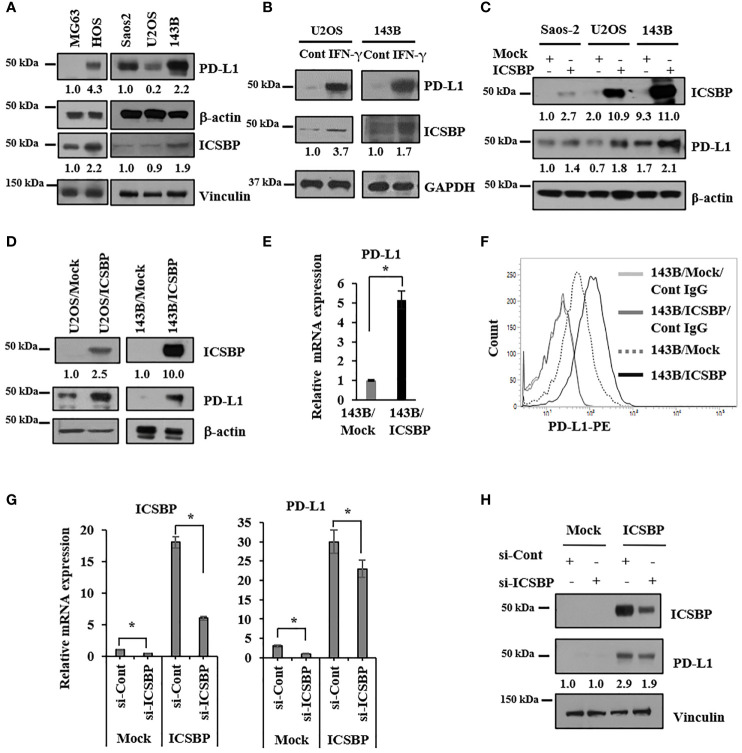
PD-L1 is induced by ICSBP in osteosarcoma cells. **(A**) Saos2, U2OS, and 143B cells were lysed, and equal aliquots of protein (20 μg) were processed for immunoblot analysis using the indicated antibodies. β-Actin and vinculin were used as loading controls. (**B**) 143B and U2OS cells were treated with 400 IU/ml of interferon-γ (IFN- γ) for 24 h and then analyzed by immunoblotting using indicated antibodies. **(C)** Saos2, U2OS, and 143B cells were transfected with either empty vector (Mock) or ICSBP-expression vector (ICSBP), followed by immunoblot analysis using indicated antibodies. **(D)** ICSBP was stably expressed in U2OS and 143B cells as described in before (CDD), and these cells were processed for immunoblot analysis using indicated antibodies. **(E)** Total RNA was isolated from 143B-Mock and 143B-ICSBP cells, and the mRNA levels of PD-L1 were assessed by real time-PCR analysis. Error bars represent standard deviations of the mean of three measurements (*p < 0.05). **(F)** 143B-Mock and 143B-ICSBP cells were stained with PE-conjugated IgG and PE-conjugated anti-PD-L1 antibodies, followed by flow cytometry analysis. **(G)** 143B-Mock and 143B-ICSBP cells were transfected with control siRNA (si-Cont) or ICSBP-specific siRNA (si-ICSBP) for 24 h, followed by real time-PCR analysis for mRNA levels **(H)** and processed for immunoblot analysis using indicated antibodies. Similar results were observed in three independent experiments.

We previously demonstrated that ICSBP promotes osteosarcoma malignancy by enhancing transforming growth factor-β (TGF-β) signaling (19, 20). Because both ICSBP and PD-L1 are induced by IFN-γ (8, 17), we explored a possible association between these two molecules in five human osteosarcoma cell lines: MG63, HOS, Saos2, U2OS, and 143B. We observed an expression of both ICSBP and PD-L1 in malignant HOS cells (24), but not in MG63 cells (**Figure 1A**). PD-L1 and ICSBP were detected in Saos2, U2OS, and 143B but were more abundant in the highly metastatic cell line 143B than in the other Saos2 and U2OS cell lines (**Figure 1A**). To assess whether IFN-γ induces ICSBP and PD-L1 expression in osteosarcoma cells, we applied IFN-γ to U2OS and 143B cells, which express relatively low and high levels of ICSBP, respectively. ICSBP and PD-L1 expressions were induced by IFN-γ treatment in both cell lines (**Figure 1B**). We next tested whether ICSBP affects PD-L1 in the absence of IFN-γ by transiently transfecting *ICSBP* into all three cell lines and assessing PD-L1 expression. ICSBP overexpression enhanced the PD-L1 expression in all three cell lines, compared to transfection of cells with a control vector (Mock) (**Figure 1C**). Similar results were obtained in U2OS and 143B cells with a stable ICSBP overexpression at the protein (**Figure 1D**) and mRNA levels in 143B cells (**Figure 1E**) and cell-surface expression levels of PD-L1 in 143B cells (**Figure 1F**). Conversely, in 143B cells stably overexpressing ICSBP (ICSBP cells), ICSBP knockdown downregulated PD-L1 at both mRNA and protein levels (**Figures 1G, H**). These data indicate that PD-L1 is induced by ICSBP in osteosarcoma cells.

### Osteosarcoma growth is suppressed by PD-L1 knockdown

**Figure 2:**
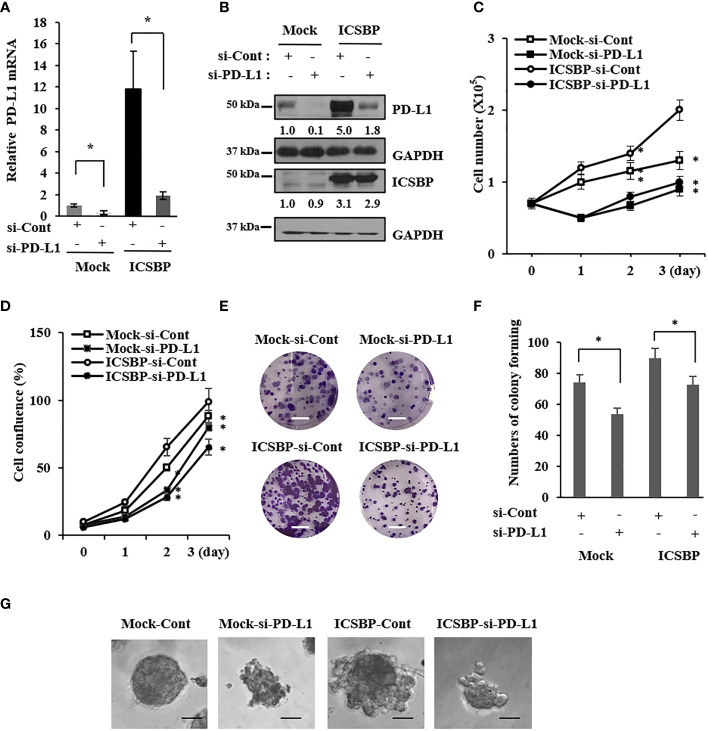
Osteosarcoma growth is suppressed by PD-L1 knockdown. 143B-Mock and 143B-ICSBP cells were transfected with control siRNA (si-Cont) or PD-L1-specific siRNA (si-PD-L1) for 24 h, followed by **(A)** real time-PCR analysis for PD-L1 mRNA levels **(B)** and immunoblot analysis using indicated antibodies. 143B-Mock and 143B-ICSBP cells were transfected with si-RNAs described above, and cell growth was analyzed **(C)** by either cell counting or **(D)** Incucyte™ system for indicated times. **(E)** 143B-Mock and 143B-ICSBP cells were transfected with si-RNAs for 24 h, and cells were plated at 1 × 10^3^ cells in a six-well plate and cultured for 7 days for colony-forming assay. Scale bar = 10 mm. **(F)** Crystal violet-stained colonies were counted (*p < 0.05). **(G)** 143B-Mock and 143B-ICSBP cells were transfected with si-RNAs for 24 h, and cells (2 × 10^3^) in 300 μl of 5% Matrigel were plated in the Matrigel-coated eight-well chamber, as described in the “**Materials and methods**” section. 3D cell images were captured after 8 days of incubation. Scale bar = 100 μm. Similar results were observed in three independent experiments. Error bars represent standard deviations of the mean of three measurements.

We previously reported that ICSBP enhances osteosarcoma cell growth and tumorigenicity (20). Because ICSBP upregulated PD-L1 expression, we examined whether PD-L1 also regulates osteosarcoma cell growth. We transfected ICSBP cells with two small interfering RNAs (siRNAs) targeting PD-L1(si-PD-L1) and observed that siRNA#2 had a stronger knockdown effect; therefore, this construct was used for all further assays (**Supplementary Figure 1**). Transfection with si-PD-L1 decreased PD-L1 mRNA and protein levels (**Figures 2A, B**). PD-L1 knockdown attenuated cell growth in both Mock and ICSBP cells, as assessed by cell counting (**Figure 2C**) and cell confluence assays using the Incucyte™ system (**Figure 2D**). In addition, PD-L1 knockdown attenuated the colony-forming capacity of 143B Mock and ICSBP cells, as determined by colony sizes (**Figure 2E**) and numbers (**Figure 2F**). PD-L1 knockdown also attenuated osteosarcoma cell growth in a three-dimensional (3D) culture (**Figure 2G**). These results indicate that PD-L1 is associated with osteosarcoma cell growth.

### PD-L1 knockdown induces apoptosis and dysregulates the cell cycle

**Figure 3:**
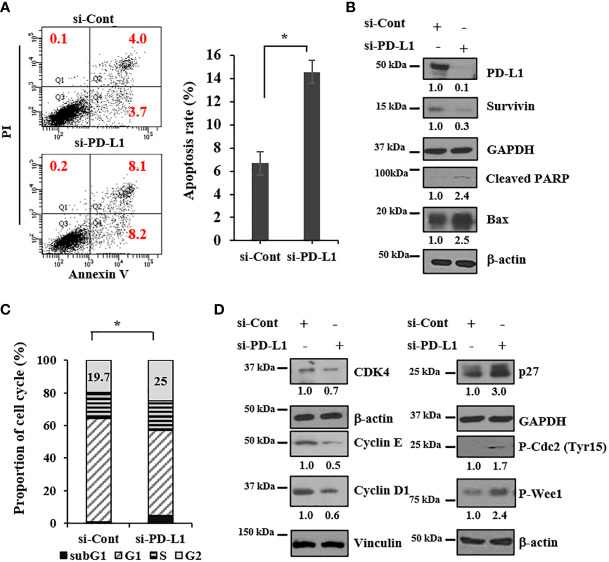
PD-L1 knockdown induces apoptosis and dysregulates the cell cycle. 143B-ICSBP cells were transfected with either si-Cont or si-PD-L1, and **(A)** cells were stained with anti-annexin-V and PI followed by flow cytometric analysis of apoptosis. The percentage of apoptotic cells is the sum of the percentage of annexin V+/PI- and double-positive cells. **(B, D)** Transfected cells were processed for immunoblot analysis using the indicated antibodies. **(C)** Transfected cells were stained with PI followed by flow cytometric analysis for cell-cycle analysis, and cell-cycle distribution is represented as a histogram. Error bars represent standard deviations of the mean of three measurements (*p < 0.05). Similar results were observed in three independent experiments.

To investigate whether growth inhibition induced by PD-L1 knockdown is caused by cell death, we performed apoptosis analysis with annexin V/propidium iodide-stained cells using flow cytometry. The apoptotic population increased by >2 fold upon PD-L1 knockdown in 143B ICSBP cells (**Figure 3A**). In addition, PD-L1 knockdown decreased the expression of survivin, an antiapoptotic protein, but increased the expression of cleaved PARP and Bax, pro-apoptotic proteins (**Figure 3B**). Because PD-L1 expression can be regulated by the cyclin D-CDK4 axis in colon cancer (25), we examined whether PD-L1 knockdown affects the cell cycle. Cell-cycle analysis revealed that the sub-G1 population was increased in ICSBP cells upon PD-L1 knockdown, indicating that cell death occurred. Moreover, PD-L1 knockdown decreased the G1 population but increased the G2 population, indicating that G2/M arrest occurred (**Figure 3C**). Interestingly, PD-L1 knockdown increased phosphorylated Cdc2 and phosphorylated Wee1, which are important for G2/M arrest (**Figure 3D**). However, PD-L1 knockdown decreased CDK4, cyclin E, and cyclin D1 but increased p27 although the G1 population was decreased (**Figure 3D**). Consistent with these results in 143B ICSBP cells (**Figure 3**), PD-L1 knockdown in U2OS ICSBP cells also induced apoptosis (**Supplementary Figure 2A**), decreased the survivin levels (**Supplementary Figure 2B**), induced G2/M arrest (**Supplementary Figure 2C**), and altered the expression of cell cycle-associated molecules (**Supplementary Figure 2D**). These results indicate that PD-L1 knockdown decreased cell growth due to cell-cycle dysregulation and apoptosis in osteosarcoma cells.

### Tumor growth is suppressed by PD-L1 knockdown in a mouse xenograft model

**Figure 4:**
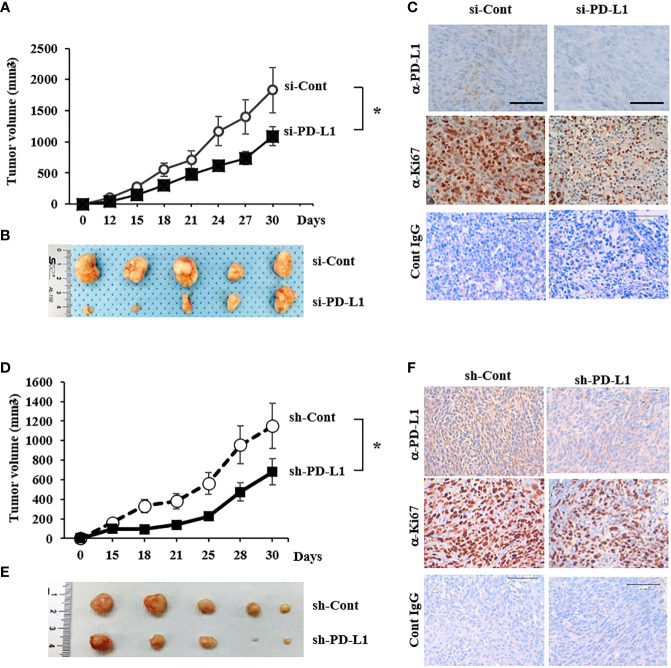
Tumor growth was suppressed by PD-L1 knockdown in a mouse xenograft model **(A)** 143B-ICSBP cells were transfected with either si-Cont or si-PD-L1 and injected subcutaneously into nude mice (n = 5), and tumor growth was monitored. Tumor size was measured with a caliper at the indicated times (*p < 0.05). **(B)** After sacrificing the mice, tumor tissues were taken, and representative images of tumors were captured. **(C)** Tumor tissues from **(B)** were stained using indicated antibodies for immunohistochemistry, and control IgG was used as a negative staining control. Scale bar, 100 μm. **(D)** 143B-ICSBP cells were stably transfected with either control vector (sh-Cont) or specific shRNA for PD-L1 (sh-PD-L1) and were injected subcutaneously into nude mice (n = 5), and tumor growth was monitored. Tumor size was measured with a caliper at the indicated times (*p < 0.05). **(E)** After sacrificing the mice, tumor tissues were taken, and representative images of tumors were captured. **(F)** Tumor tissues were stained using indicated antibodies for immunohistochemistry, and control IgG was used as a negative staining control. Scale bar, 100 μm. Error bars represent standard deviations of the mean of three measurements (*p < 0.05). Similar results were observed in three independent experiments.

To explore the effect of PD-L1 knockdown on tumor growth *in vivo*, 143B ICSBP cells, transfected with either a control siRNA (si-Cont) or si-PD-L1, were injected subcutaneously into Balb/c nude mice to build a xenograft model. Tumor growth was suppressed considerably by PD-L1 knockdown (**Figures 4A, B**). Tumors were extracted from the sacrificed mice and processed for immunohistochemical analysis. The expression of both PD-L1 and Ki67 (proliferation marker) was reduced in si-PD-L1 tumor specimens as compared with that in si-Cont tumor specimens (**Figure 4C**). The same experiment was conducted using ICSBP cells stably transfected with either a control short hairpin RNA (sh-Cont) or a PD-L1-specific short hairpin RNA (sh-PD-L1) vector. Of the two sh-PD-L1 clones, sh-PD-L1 #2 had a stronger knockdown effect and we used it for further experiments (**Supplementary Figure 3**). We observed that transfection with sh-PD-L1 decreased both PD-L1 mRNA levels and the surface expression of PD-L1 protein in ICSBP cells (**Supplementary Figures 3B, C**). Similar results to those shown in **Figure 4A–C** were obtained in terms of growth suppression (**Figures 4D–F**) and reduction of PD-L1 and Ki-67 expression in sh-PD-L1 tumors (**Figure 4F**). These data suggest that PD-L1 contributes to the growth of 143B ICSBP cells.

### PD-L1 knockdown and doxorubicin treatment synergistically inhibit the growth of osteosarcoma cells

**Figure 5:**
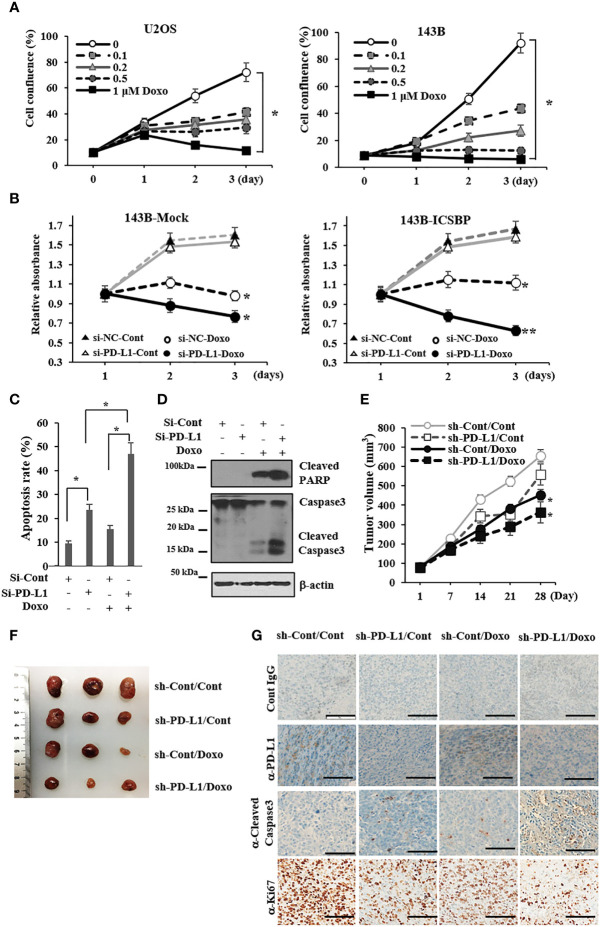
PD-L1 knockdown and doxorubicin treatment synergistically inhibit the growth of osteosarcoma cells. **(A)** U2OS and 143B cells (5 × 10^3^ cells/well) were seeded and treated with indicated concentrations (0–1μM) of doxorubicin for indicated times, and cell proliferation was measured with the Incucyte™ system (*p < 0.05). **(B)** 143B-Mock and 143B-ICSBP cells were transfected with either si-Cont or si-PD-L1 24 h, and cells were treated without or with 0.2 μM doxorubicin for indicated times, followed by MTS assay for cell growth (*p <0.05). **(C, D)** 143B-ICSBP cells were transfected with either si-Cont or si-PD-L1 for 24 h and treated with doxorubicin (0.2 μM) for 24 h, followed by **(C)** apoptosis assay or **(D)** immunoblotting. For apoptosis assay, cells were stained with annexin V-FITC and propidium iodide (PI), followed by flow cytometry analysis. The percentage of apoptotic cells is the sum of the percentage of annexin V+/PI- and double-positive cells. Data are reported as means ± SD for three independent experiments (p < 0.05). **(E-G)** Established 143B-ICSBP cells that express control vector (sh-Cont) or specific shRNA for PD-L1 (sh-PD-L1) were injected subcutaneously into nude mice (n = 5).When the tumor size reached 30 mm^3^, tumor-bearing mice received intraperitoneal injection with either PBS or 5 mg/kg of doxorubicin twice a week for 4 weeks. At the indicated times, tumor volumes were measured with a caliper **(E)**. Significant difference (*p < 0.05) in the terminal tumor volumes between control and the combination treatment group was analyzed by ANOVA. **(F)** After sacrificing the mice, tumors were taken and processed for tumor imaging and **(G)** tumor tissues from **(F)** were stained using indicated antibodies for immunohistochemistry, and control IgG was used as a negative staining control. Scale bar, 100 μm. Similar results were observed in two independent experiments with error bars representing the standard deviation.

Doxorubicin is one of the most active drugs against osteosarcoma (26). We evaluated the effect of doxorubicin on the growth of U2OS and 143B cells. Doxorubicin inhibited cell growth in both cell lines in a dose-dependent manner, and its inhibitory effect was somewhat greater in 143B cells than in U2OS cells (**Figure 5A**). Because PD-L1, which is associated with chemotherapy resistance (27), was induced by ICSBP (**Figure 1**), we explored the influence of ICSBP on si-PD-L1 and/or doxorubicin-mediated growth inhibition using 143B Mock and ICSBP cells. Doxorubicin hindered the growth of both cells (**Figure 5B**). Doxorubicin suppressed growth more profoundly in si-PD-Ll cells than in si-Cont cells. This synergistic effect of si-PD-L1 and doxorubicin on growth inhibition seemed to be slightly greater in ICSBP cells than in Mock cells. Next, we tested whether apoptosis was involved in the synergistic growth inhibition of ICSBP cells. When PD-L1 knockdown was combined with doxorubicin treatment, the apoptotic rate increased to 47% from 23.5% with PD-L1 knockdown alone and 15.4% with doxorubicin treatment alone (**Figure 5C**). Moreover, doxorubicin-induced-caspase 3 activation was substantially increased by PD-L1 knockdown as reflected by increased PARP cleavage and cleaved caspase 3 (**Figure 5D**). To reenact synergistic growth inhibition *in vivo*, either ICSBP-sh-Cont cells or ICSBP-sh-PD-L1 cells were injected into mice subcutaneously, and the mice were given doxorubicin. To our expectation, synergistic growth inhibition was observed in the sh-PD-L1 group treated with doxorubicin (**Figures 5E, F**). Removed tumor tissues were subjected to immunohistochemical analysis, which showed that the expression of a cell proliferation maker, Ki-67, was lower in sh-PD-L1 mice treated with doxorubicin than in mice that received either the sh-PD-L1-alone or doxorubicin-alone group (**Figure 5G**). The expression of the apoptosis marker cleaved caspase 3 was also higher in sh-PD-L1 mice treated with doxorubicin than in mice that received either sh-PD-L1 alone or doxorubicin alone (**Figure 5G**). Taken together, these results indicate that the combination of PD-L1 knockdown and doxorubicin treatment exerts a synergistic inhibitory action on ICSBP cells.

### ICSBP and PD-L1 expression are correlated in human osteosarcoma tissues

**Figure 6:**
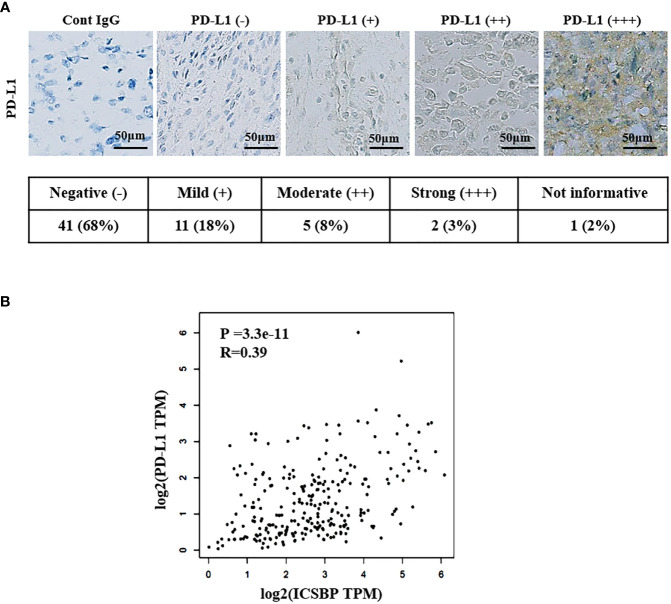
ICSBP and PD-L1 expressions are correlated in human osteosarcoma tissues. **(A)** Patient-derived osteosarcoma tissue microarray was subjected to immunohistochemistry using anti-PD-L1 antibodies. Staining results were graded according to the intensity and proportion of positive cells as described in the “**Materials and methods”**. **(B)** Correlation of ICSBP and PD-L1 mRNA expression was analyzed by GEPIA2 from TCGA sarcoma tumor data set using the Spearman’s correlation method (r = 0.39, p = 3.3e-11).

We examined PD-L1 expression in osteosarcoma patient samples by immunostaining. The staining intensity of PD-L1 varied from negative (–) to strong (+++), as depicted in **Figure 6A** and **Supplementary Table 1**. Among the 59 informative specimens, 30.5% (18/59) were PD-L1-positive and 69.5% (41/59) were PD-L1-negative (**Figure 6A**). We previously demonstrated that 83% (45/54) of the evaluable specimens from the same tissue microarray were ICSBP-positive (19). We then utilized Gene Expression Profiling Interactive Analysis2 (GEPIA2) to examine the co-expression of ICSBP and PD-L1 in The Cancer Genome Atlas sarcoma data (N = 264), a larger sample. Although weak, PD-L1 expression was positively correlated with ICSBP expression (*R* = 0.39; *P* = 3.3e-11) as shown in **Figure 6B**. These data indicate that ICSBP and PD-L1 expression is associated not only in osteosarcoma cells *in vitro* but also in human osteosarcoma tissues.

## Discussion

We previously reported that ICSBP expression contributes to malignant phenotypes and EMT by activating the TGF-β receptor pathway in U2OS and 143B human osteosarcoma cells (19, 20). Many studies have demonstrated that PD-L1 expression in cancer cells is associated with tumor malignancy (28). In the current study, we first demonstrated that ICSBP expression increased PD-L1 expression in osteosarcoma cells and that PD-L1 expression was associated with osteosarcoma growth *in vitro* and *in vivo*.

PD-L1 is regulated by various molecules, such as STAT3 in T-cell lymphoma and melanoma (29), STAT and AP-1 in classical Hodgkin lymphoma (30), the ZEB1 axis in non-small-cell lung cancer (31), PI3K in glioma (32), and Myc in multiple tumors (33). IFN-γ upregulates PD-L1 through the JAK2/STAT1/IRF-1 axis (34). IFN-γ induces the expression of IRFs, which include ICSBP (IRF8) (16). Of the three osteosarcoma cell lines we used, highly metastatic 143B cells showed the highest expression of both ICSBP and PD-L1 (**Figure 1A**). These data suggest that ICSBP expression is associated with PD-L1 levels. IFN-γ treatment of U2OS and 143B cells enhanced the PD-L1 expression along with inducing ICSBP (**Figure 1B**). ICSBP knockdown attenuated the induction of PD-L1 expression by IFN-γ treatment, although it did not alter STAT1 phosphorylation induced by IFN-γ (**Supplementary Figure 4**). In addition, ICSBP overexpression itself upregulated PD-L1 without IFN-γ treatment (**Figure 1C-G**) and ICSBP knockdown downregulated PD-L1 (**Figure 1H**), indicating that ICSBP can regulate PD-L1 levels independently of IFN-γ.

Growth inhibition and suppression of colony formation by PD-L1 knockdown were more pronounced in ICSBP cells than in Mock cells (**Figure 2**). These results indicate that progression of tumor cells with ICSBP overexpression seems to be more effectively blocked by PD-L1 knockdown. Apoptosis and cell-cycle dysregulation were elicited by PD-L1 knockdown in ICSBP cells (**Figure 3**), such that the sub-G1 and -G2 proportions increased but the G1 proportion decreased (**Figure 3C**). PD-L1 knockdown increased p27, which arrests G1, S, and G2/M phases (35) but decreased the levels of G1 phase molecules, such as cyclins D1, cyclin E, and CDK4 (**Figure 3D**). Additionally, phosphorylation of both Cdc2 and Wee1 was increased; these molecules are involved in G2/M arrest (**Figure 3D**). Accordingly, PD-L1 knockdown increased the G2 population, probably due to G2/M arrest. However, PD-L1 knockdown did not increase the G1 population despite decreased levels of G-phase cyclins. This reduction in the G1 population could be due to increased G2/M arrest and the increased sub-G1 population (**Figure 3C**). Apoptosis and cell-cycle arrest induced by PD-L1 knockdown were not unique to 143B ICSBP cells because we observed very similar phenomena in U2OS ICSBP cells by PD-L1 knockdown (**Supplementary Figure 2**).

The most effective chemotherapeutic agents in osteosarcoma include doxorubicin, methotrexate, and cisplatin (26). We examined the growth inhibitory effect of doxorubicin on two osteosarcoma cell lines, 143B and U2OS, which express different levels of PD-L1 and ICSBP (**Figure 1A**). We found that 143B cells with higher levels of PD-L1 and ICSBP were more sensitive to doxorubicin-induced cell death than U2OS cells with lower levels of PD-L1 and ICSBP (**Figure 5A**). PD-L1 induction by IFN-γ can confer resistance to doxorubicin in metastatic breast cancer MDA-MB-231 cells (27), which contrasts with our finding. The difference in sensitivity could be due to differences in PD-L1 expression state, such as the transient induction of PD-L1 by IFN-γ in MDA-MB-231 cells (27) versus the intrinsic PD-L1 expression in 143B cells in our study (**Figure 1A**).

We explored whether PD-L1 knockdown could accentuate the growth inhibitory effect of doxorubicin in ICSBP cells. PD-L1 knockdown alone slightly suppressed growth in ICSBP cells (**Figure 5B**). However, PD-L1 knockdown combined with doxorubicin treatment suppressed the growth of ICSBP cells to a greater extent than did doxorubicin treatment alone, indicating that the combination has synergistic effects (**Figure 5B, E, F**). These data are consistent with a recent report demonstrating that the combination of checkpoint blockade and doxorubicin augments apoptosis in osteosarcoma (36). We also examined the combination of PD-L1 knockdown with other chemotherapeutic agents, methotrexate and cisplatin. PD-L1 knockdown combined with each of these agents synergistically induced apoptosis, indicating that the synergistic effect of PD-L1 combination therapy is not limited to doxorubicin, although PD-L1 knockdown plus doxorubicin induced apoptosis more effectively than did the other combinations (**Supplementary Figure 5**).

To exclude the possibility that the antitumor effects of PD-L1 knockdown and/or doxorubicin treatment are due to transfection and/or establishment of a stable cell line, we evaluated apoptosis induced by PD-L1 knockdown in 143B parental cells, which express higher levels of both ICSBP and PD-L1 than any other osteosarcoma cell lines (**Figure 1A**). Either PD-L1 knockdown or doxorubicin treatment enhanced the apoptosis of 143B parental cells, and the combination of the two showed additive effects (**Supplementary Figure 6**), although these effects were less marked than those in ICSBP cells (**Figure 5C**), which is similar to the effects seen in the 143B Mock and 143B ICSBP cells (**Figure 5B**). It has been reported that PD-L1 expression varies among different osteosarcoma tissues (37). In agreement with that report, only 30% of the samples in the tissue array were positive for PD-L1 expression and the intensity of expression was variable (**Figure 6A** and **Supplementary Table 1**). These findings suggest that the synergistic action of PD-L1 knockdown and doxorubicin treatment might inhibit in a certain fraction of osteosarcoma patients, but not in all.

It has been reported that coculture of PD-1-overexpressing lymphocytes with PD-L1 overexpressing osteosarcoma cells results in osteosarcoma cell proliferation (38). The anti-PD-1 antibody synergizes with cisplatin to promote osteosarcoma cell apoptosis *in vitro* and to reduce tumor volume *in vivo* (38). Although we did not explore a role for PD-1 in osteosarcoma cell growth *in vitro*, it is possible that PD-L1 on osteosarcoma cells interacts with PD-1 on lymphocytes in the tumor microenvironment which could enhance tumor growth *in vivo*. In addition, a recent report revealed that PD-1 was expressed in the osteosarcoma microenvironment and PD-L1 was expressed in osteosarcoma cells in a biopsy sample (39). These studies indicate that targeting the PD-1/PD-L1 axis and chemotherapy could be a useful strategy to control osteosarcoma.

In summary, the current study showed that PD-L1 was induced by ICSBP and that PD-L1 and ICSBP were involved in growth and tumorigenicity of osteosarcoma cells. PD-L1 knockdown and doxorubicin treatment synergistically inhibited the growth of osteosarcoma cells carrying ICSBP and PD-L1 proteins. This synergistic effect is worth investigating in an osteosarcoma clinical trial, considering that osteosarcoma survival with chemotherapy alone has not improved over the last several decades (40).

## Data availability statement

The raw data supporting the conclusions of this article will be made available by the authors, without undue reservation.

## Ethics statement

The Animal study was conducted in accordance with the Institutional Animal Care and Use Committee (IACUC) of National Cancer Center Research Institute (NCCRI) and IACUC approval number is NCC-16-152C.

## Author contributions

JYS; performed experiments, analyzed data, and wrote the manuscript. JHK, HGK and JWP; collected tissue samples and advised for tissue experiments. SYP; performed pathological scoring and pathological experiments. BKP and YNK; wrote the manuscript and supervised the project. All authors contributed to the article and approved the submitted version.

## Funding

This study was supported by a research grant from the National Cancer Center, Korea (2110310), National Research Foundation (NRF) grant funded by the Korea government (2017R1D1A1B03034), and the Korean Foundation for Cancer Research (1732050).

## Acknowledgments

We thank the Research Core Center in National Cancer Center Korea for assistance with our experiments. We thank the Laboratory Animal Research Facility in National Cancer Center Korea, Dr. Mi Sun Park and Hee Seo Park for the supporting experiments, and Tae Sik Kim for assisting with flow cytometry experiments and analysis.

## Conflict of interest

The authors declare that the research was conducted in the absence of any commercial or financial relationships that could be construed as a potential conflict of interest.

## Publisher’s note

All claims expressed in this article are solely those of the authors and do not necessarily represent those of their affiliated organizations, or those of the publisher, the editors and the reviewers. Any product that may be evaluated in this article, or claim that may be made by its manufacturer, is not guaranteed or endorsed by the publisher.
