# Supplementary material for: ICSBP-induced PD-L1 enhances osteosarcoma cell growth
Source: Front Oncol. 2022 Sep 23;12:918216. doi: 10.3389/fonc.2022.918216 (PMC9555079; doi:10.3389/fonc.2022.918216)
Supplement: Supplementary file 1 [file DataSheet_1.docx]

**Supplementary Data**

**Supplementary Figure 1**. **Validation of si-PD-L1s in 143B osteosarcoma cells.** 143B Mock and ICSBP overexpressing cells were transiently transfected with either si-Cont or si-PD-L1 for 24h, cells were then processed for immunoblot analysis using indicated antibodies. β-actin was used as a loading control. These experiments were performed two times independently with similar results.

**Supplementary Figure 2**. **PD-L1 knockdown induces apoptosis and dysregulates cell cycle in U2OS cells.** U2OS-ICSBP cells were transfected with either si-Cont or si-PD-L1 and **(A)** cells were stained with anti-annexin-V and PI and followed by flow cytometric analysis of apoptosis. The percentage of apoptotic cells is the sum of the percentage of annexin V+/PI- and double-positive cells. **(B and D)** Transfected cells were processed for immunoblot analysis using the indicated antibodies. **(C)** Transfected cells were stained with PI followed by flow cytometric analysis for cell cycle analysis and cell-cycle distribution is represented as a histogram. Error bars represent standard deviations of the mean of three measurements (* p< 0.05). Similar results were observed in three independent experiments.

**Supplementary Figure 3**. **Establishment of sh-PD-L1 stable cell line**. 143B-ICSBP cells were transfected with sh-PD-L1 lentiviral particle as described in the “**Materials and Methods**”. **(A)** Stably expressed sh-Control (si-Cont) and sh-PD-L1 cell clone (sh-PD-L1-1 and 2) were performed with immunoblot analysis using indicated antibodies. β-actin was used as a loading control. **(B)** sh-Cont and sh-PD-L1 cells were lysed and followed by real time-PCR analysis for mRNA levels. **(C)** sh-Cont and sh-PD-L1 cells were stained with PE-conjugated IgG and PE-conjugated anti-PD-L1 antibodies, followed by flow cytometry analysis **(D)** Tumor tissues from sh-PD-L1 injected mouse were taken and analyzed expression with immunoblotting assay. These experiments were performed two times independently with similar results.

**Supplementary Figure 4.** **Interferon-γ effect by ICSBP knock down. (A)**143B cells were transfected with control siRNA (si-Cont) or ICSBP-specific siRNA (si-ICSBP) for 24 h. Cells were treated interferon-γ (IFN- γ) for 24 hrs. Cells were analyzed immunoblotting assay using indicated antibodies. These experiments were performed two times independently with similar results.

**Supplementary Figure 5.** **Combination effects with knock down of PD-L1 and chemotherapeutic drugs.**143B-ICSBP cells were transfected with either si-Cont or si-PD-L1 and cells were and treated with MTX (1μM), and Cisplatin (5μM) for 24h, followed by apoptosis assay. **(A)** For apoptosis assay, cells were stained with anti-annexin-V and PI and followed by flow cytometric analysis of apoptosis. **(B)** Cells were and treated with doxorubicin (0.2μM), MTX (1μM), and Cisplatin (5μM) for 24h. Apoptotic cells were quantified with early and late apoptotic cell populations with flow cytometric analysis These experiments were performed two times independently with similar results.

**Supplementary Figure 6. Apoptosis induction by knock down of PD-L1 and doxorubicin combinations.** 143B cells were transfected with either si-Cont or si-PD-L1 and cells were and treated with doxorubicin (0.2μM) for 24h, followed by apoptosis assay. **(A)** For apoptosis assay, cells were stained with anti-annexin-V and PI and followed by flow cytometric analysis of apoptosis. **(B)** Apoptotic cells were quantified with early and late apoptotic cell populations with flow cytometric analysis results. These experiments were performed two times independently with similar results.

**Supplementary Table 1. Immunostaining scoring of TMA** Patient-derived osteosarcoma tissue microarray was subjected to immunohistochemistry using anti-PD-L1 antibodies. Staining results were graded according to the intensity and proportion of positive cells as described in the “**Materials and methods”**

**Supplementary Information**

**Cell lines and culture** Human osteosarcoma cell lines 143B and U2OS was obtained from the American Type Culture Collection (Rockville, MD, USA). Cells were maintained in RPMI 1640 medium (Life Technologies, Grand Island, NY, USA) supplemented with 10% fetal bovine serum (FBS; Life Technologies), 100 units/ml penicillin, and 100 μg/ml streptomycin (Life Technologies) at 37°C in a humidified incubator with an atmosphere containing 5% CO_2_.

**Establishment of ICSBP-expressing stable cell lines** ICSBP PCR product was cloned into the *Hin*dIII and *Xho*I sites of pcDNA3.1/V5-HisA vector (Invitrogen, MA, USA). 143B and U2OS cells were transfected with ICSBP construct or empty vector (Mock) using Lipofectamine 2000 (Life Technologies) according to the manufacturer’s instructions. Stable cell lines, including 143B-Mock and 143B-ICSBP cells were established by selection with 500 μg/mL of geneticin (G418, Calbiochem, La Jolla, CA, USA) for 4 weeks.

**Establishment of sh-PD-L1 cell lines** PD-L1 shRNA lentiviral transduction particles (TRCN0000002746 and TRCN0000002749) and non-targeting shRNA lentiviral transduction particles (pLKO.1-puro Non-Target Control [SHC016V]) and non-targeting shRNA lentiviral transduction particles (pLKO.1-puro Non-Target Control [SHC016V]) were obtained from OriGene. Lentiviral particles were added in cultured cells. After 48 hours, media was changed to fresh media with 2 μg/ml puromycin. Media was replaced every third day with fresh puromycin-containing media until stable clones were identified. PD-L1 knockdown was confirmed using immunoblotting analysis.

**Antibodies and reagents** Polyclonal antibodies against PD-L1, pSTAT1, vinculin, CDK4, Cyclin D1, Cyclin E, GAPDH and β-actin were purchased from were purchased from Cell Signaling Technology (Beverly, MA, USA). The ICSBP antibodies (anti-goat and anti-mouse), IRF1, and IRF4 were obtained from Santa Cruz Biotechnology (Santa Cruz, CA, USA). PD-L1-PE antibody was purchased from eBioscience (Thermo Fisher scientific, Waltham, MA, USA) and poly clonal antibody against vinculin was purchased from abcam (Cambridge, UK). Doxorubicin and methotrexate were purchased from Sigma (St. Louse, MI, USA).

**Immunoblot analyses** After washing with ice-cold PBS (10 mM Na_2_HPO_4_ pH 7.4, 145 mM NaCl, and 2.7 mM KCl), cells were lysed with 2 × sodium docecyl sulfate (SDS)-PAGE sample buffer (20 mM Tris pH 8.0, 2% SDS, 2 mM DTT, 1 mM Na_3_VO_4_, 2 mM EDTA, and 20% glycerol) and boiled for 5 min. The protein concentration of each sample was determined by using the Micro-Bicinchoninic Acid protein assay reagent as described by the manufacturer (Thermo Scientific, Rockford, IL, USA). Total cellular protein (30 μg/lane) was separated by 10% SDS-PAGE and transferred to polyvinylidene difluoride (PVDF) membranes. Membranes were blocked overnight at 4°C in TBST (20 mM Tris pH 8.0, 150 mM NaCl, and 0.05% Tween 20) containing 5% non-fat milk. Membranes were then incubated overnight at 4°C with primary antibody, washed three times with TBST, incubated with horseradish peroxidase (HRP)-conjugated goat anti-rabbit IgG secondary antibody for 1 h at room temperature, and washed three times with TBST. Proteins were visualized using an enhanced chemiluminescence reagent (Millipore).

**Co-Immunoprecipitation analyses** After washing with ice-cold PBS (10 mM Na_2_HPO_4_ pH 7.4, 145 mM NaCl, and 2.7 mM KCl), cells were lysed with RIPA buffer (25 mM Tris-HCl, pH7.5, 150 mM NaCl, 1% Nonidet P-40, 0.1% SDS, 1% sodium deoxycholate, 1 mM PMSF, protease inhibitor). Solubilized lysates were incubated with specific antibody or preimmune IgG at a final concentration of 1 µg/mL overnight at 4°C. Protein A+G-Agarose (Santa Cruz) was then added for 2 h at 4°C. The samples were centrifuged and washed three times with RIPA buffer and prepared for Western blotting by boiling in sample buffer. The immunoprecipitated proteins were detected by Western blot analysis.

**Quantitative real time PCR** Total RNA was extracted from the samples with Ribospin (GeneAll,seoul, Korea). 1 μg of total RNA was converted into cDNA by using HelixCript Easy cDNA Synthesis Kit (Nanohelix) and quantitative PCR analysis was performed on the Roche Light Cycler® 96 by SYBR-GREEN qPCR method (Nanohelix). All reactions were performed in triplicate, and the relative transcript abundance of each tested gene was normalized to the expression level of housekeeping gene. cDNA fragments were amplified using the following primer pairs: human ICSBP, 5’-GGATATGCCTATGACACA-3’ (sense) and 5’-CATCCGGCCCATACAACTTAG-3’ (anti-sense); human PD-L1, 5’-ATCACTATCCCATTAGACACATC-3’ (sense) and 5’-CAAGAAACAGTTGACTTACGATT-3’ (anti-sense); and human GAPDH, 5’-ACTCAACACGGGAAACCTCA-3’ (sense) and 5’- AACCAGACAAATCGCTCCAC-3’ (anti-sense). Relative expression was calculated by the comparative Ct method. [2(-ddCt)] of each molecule was calculated as follows: dCt = Ct (molecule) – Ct (GAPDH); ddCt = dCt (experiment or target) – dCt (control or reference).

**Flow cytometry analysis** Cells were trypsinized and suspended in PBS containing 2.5 mM EDTA, 2.5 mM EGTA and 1% BSA. For the apoptosis assay, treated cells were harvested and incubated for 15 min at RT with FITC-conjugated annexin-V reagent (2.5 μg/ml) and propidium iodide (PI) (5 μg/ml) in binding buffer followed by flow cytometer analysis. Apoptotic cell death was analyzed by the total percentage of early and late apoptotic cells in different groups. For the cell cycle analysis, cells were fixed with 70% ethanol for 2 hours at 4℃ and stained with PI solution. Cell cycle distributions were analysis with PI contents in the cells with flow cytometer analysis. The Data were analyzed with Cell Quest Software (BD bioscience, San Jose, CA). Data are means ± SD of three independent experiments.

**Immunohistochemical staining** Tumor tissues were fixed with 10% neutral buffered formalin. Formaldehyde-fixed specimens were paraffin-embedded and cut to a thickness of 4 μm. Sections were dried at 56°C for 1 h, and immunohistochemical staining was performed with Discovery XT (Ventana Medical Systems, Tucson, Arizona, USA) as follows. Sections were deparaffinized, rehydrated with EZ prep (Ventana Medical Systems), and washed with reaction buffer. Antigens were retrieved with heat treatment in Tris-ethylenediaminetetraacetic acid (EDTA) pH 8.0 buffer (CC1, Ventana Medical Systems) at 90°C for 30 min for specific antibodies, anti-PD-L1 (1:100 dilution; Cell Signaling Technology), and anti-Ki67 antibodies (1:200 dilution; Cell Signaling Technology). Parallel sections incubated with normal IgG instead of primary antibodies were used as negative controls. The overall staining results were scored from 0 to 3 based on the intensity and positive rate of staining. Intensity of staining was categorised as 0, negative ( - ); 1, weak ( + ); 2, intermediate( ++ ); 3, strong ( +++ ). Stained tissue arrays were reviewed by two experienced pathologists.


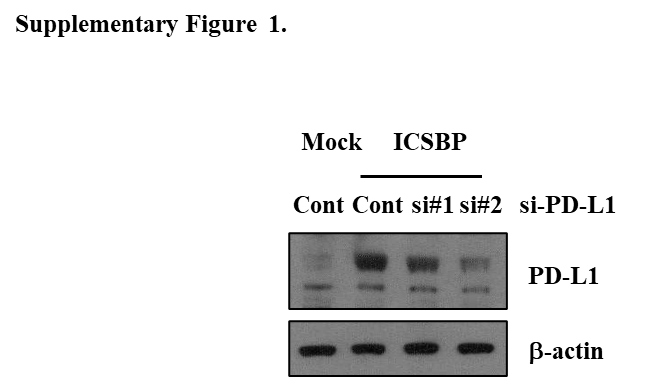


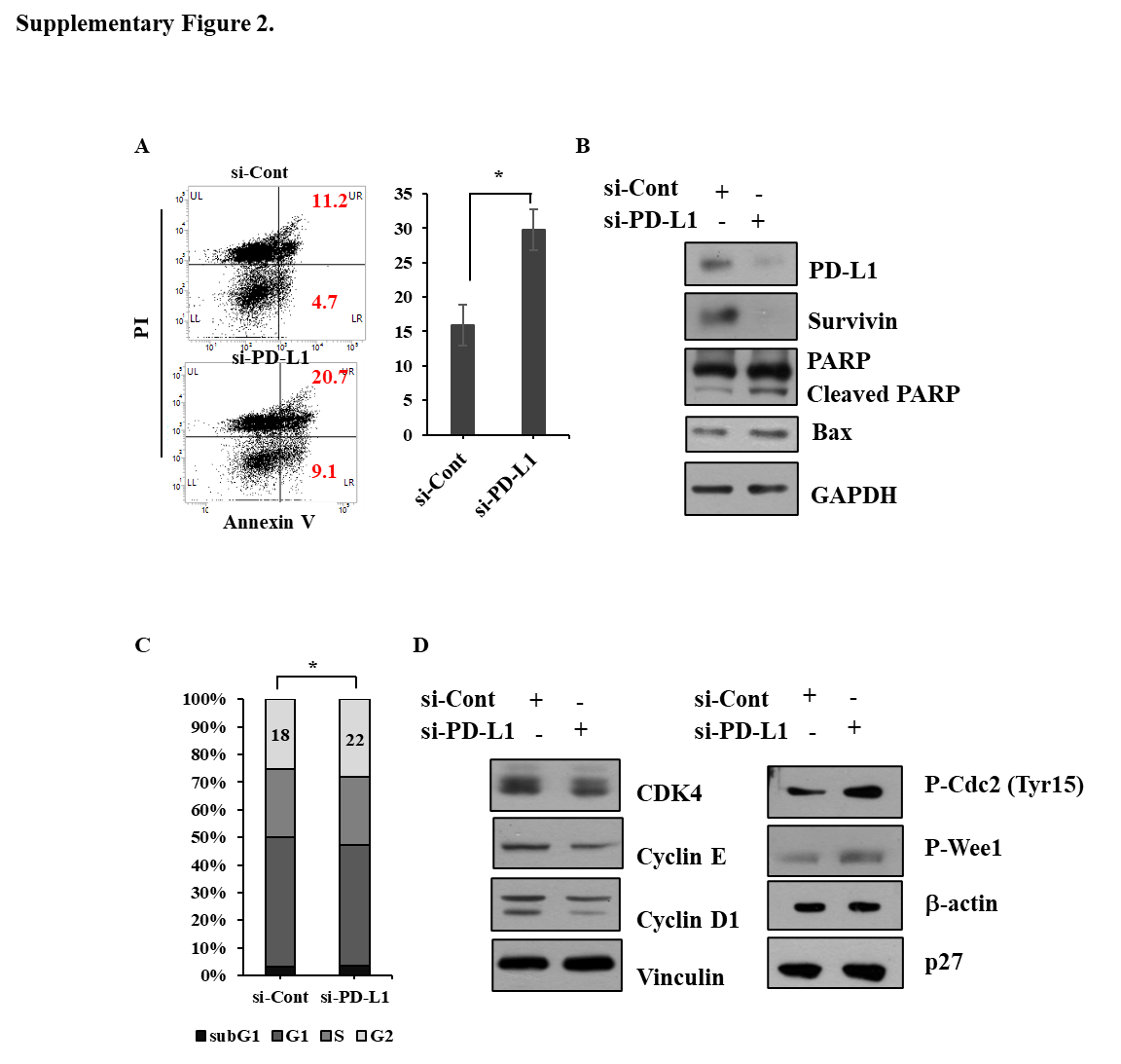


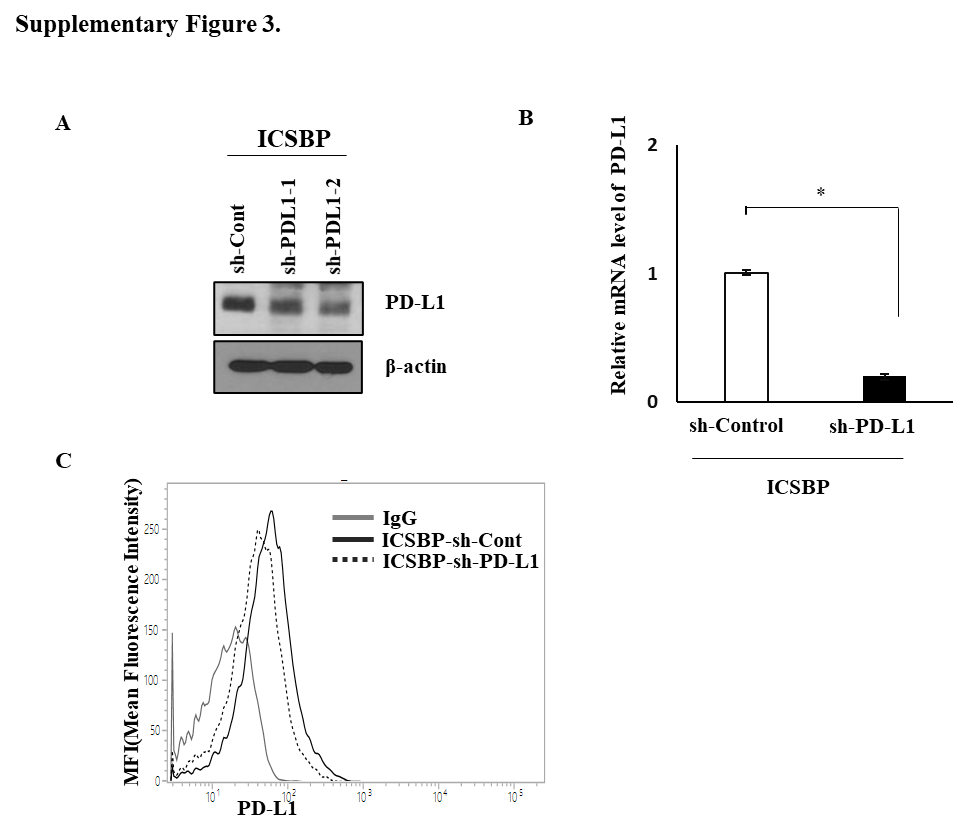


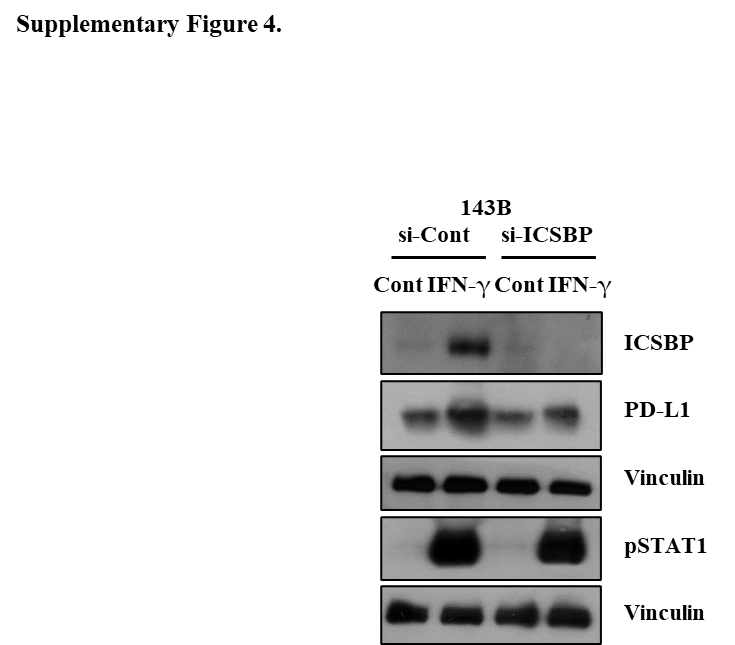


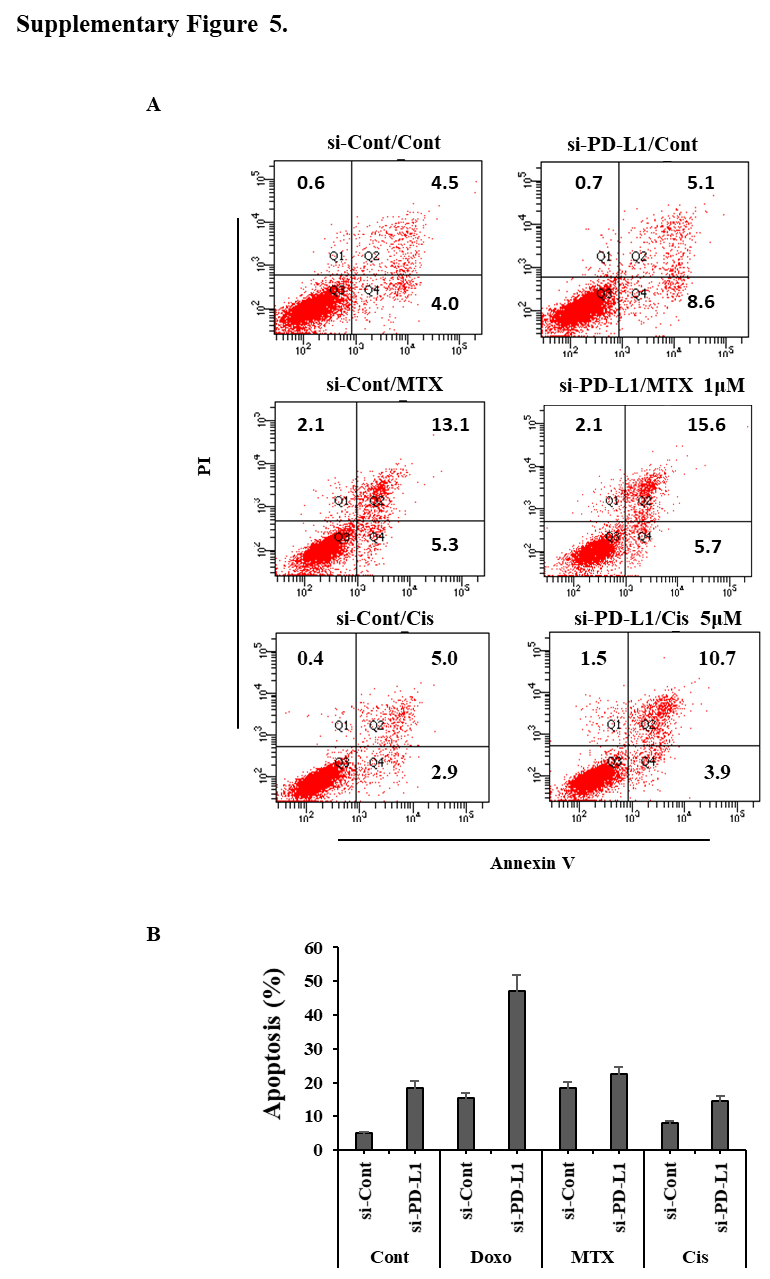


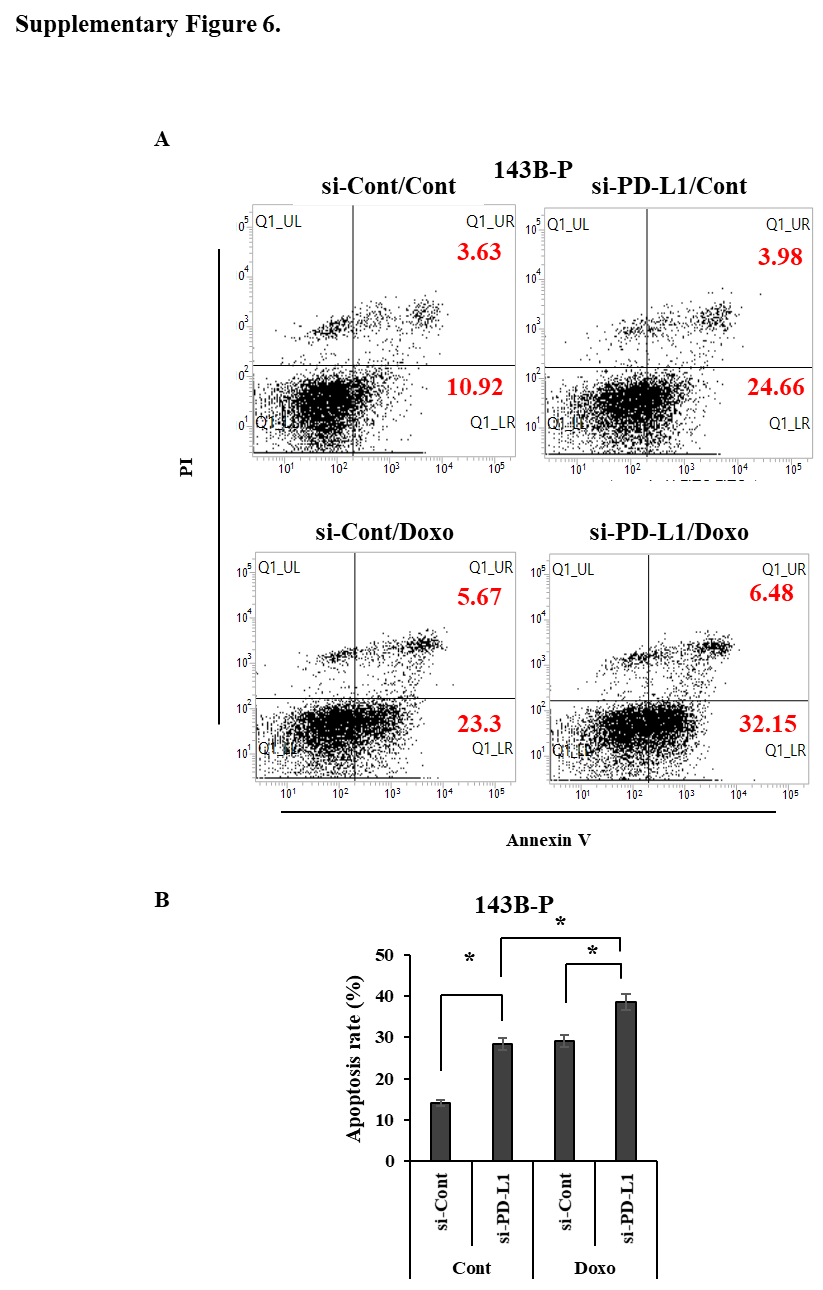


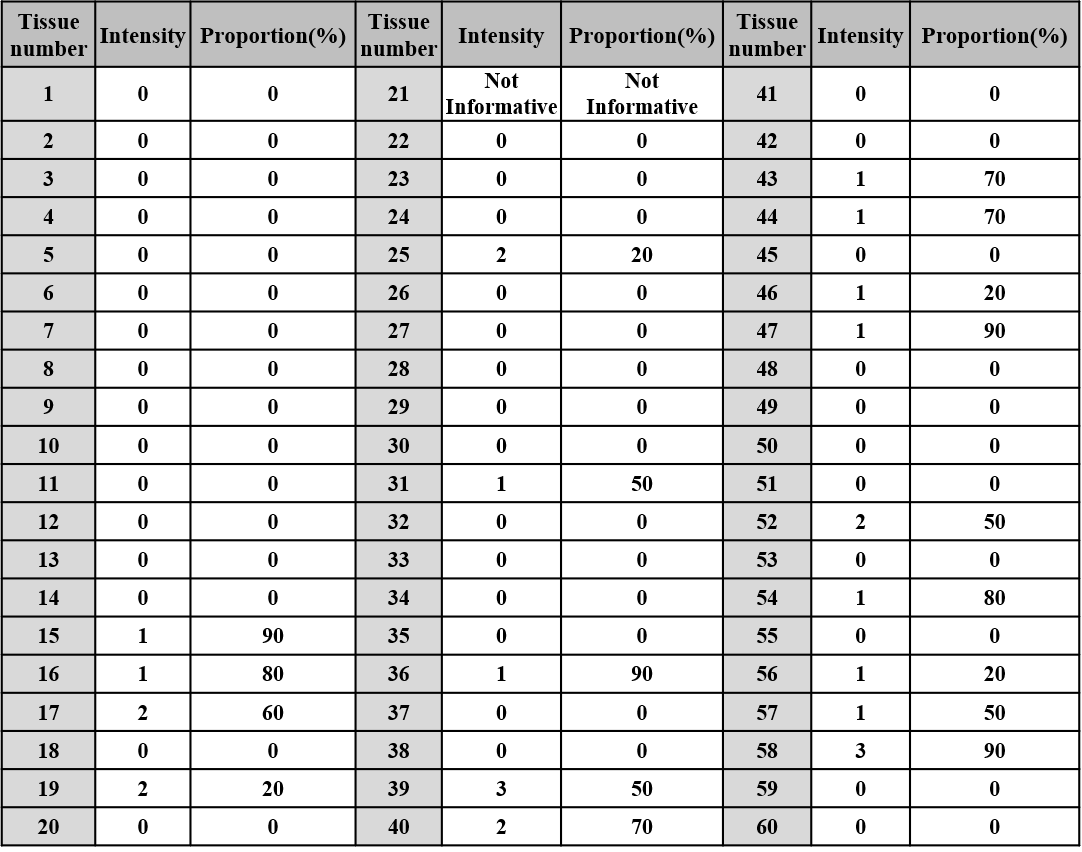


**Supplementary Table 1.**
