# Supplementary figures and images for: ICSBP-induced PD-L1 enhances osteosarcoma cell growth
Source: Front Oncol. 2022 Sep 23;12:918216. doi: 10.3389/fonc.2022.918216 (PMC9555079; doi:10.3389/fonc.2022.918216)

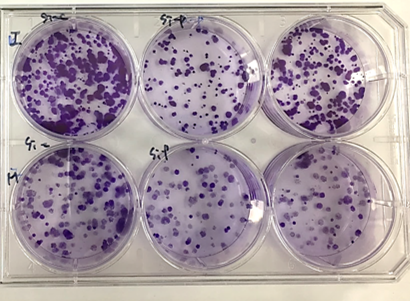

Supplement: Supplementary file 2 [file DataSheet_2.zip › Image 1.TIF]

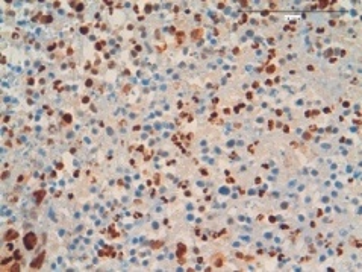

Supplement: Supplementary file 2 [file DataSheet_2.zip › Image 10.TIF]

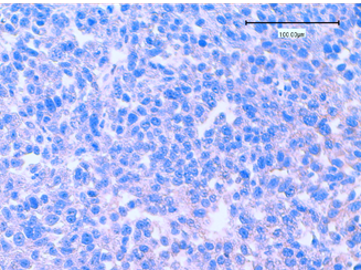

Supplement: Supplementary file 2 [file DataSheet_2.zip › Image 11.TIF]

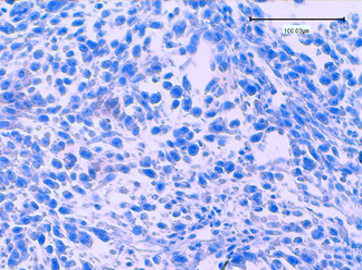

Supplement: Supplementary file 2 [file DataSheet_2.zip › Image 12.TIF]

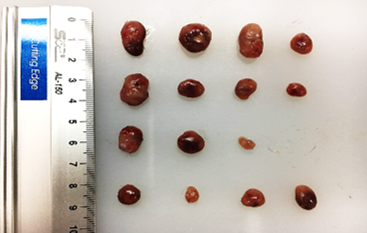

Supplement: Supplementary file 2 [file DataSheet_2.zip › Image 13.TIF]

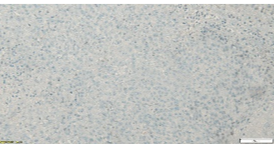

Supplement: Supplementary file 2 [file DataSheet_2.zip › Image 14.TIF]

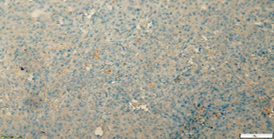

Supplement: Supplementary file 2 [file DataSheet_2.zip › Image 15.TIF]

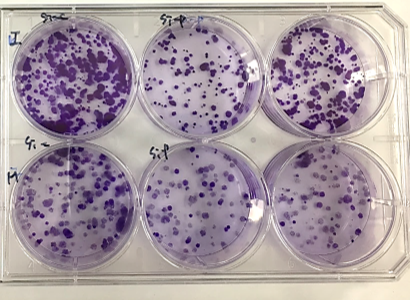

Supplement: Supplementary file 2 [file DataSheet_2.zip › Image 2.TIF]

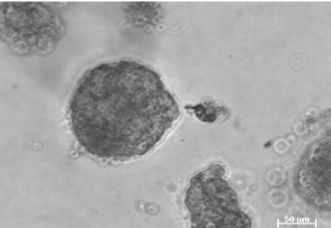

Supplement: Supplementary file 2 [file DataSheet_2.zip › Image 3.TIF]

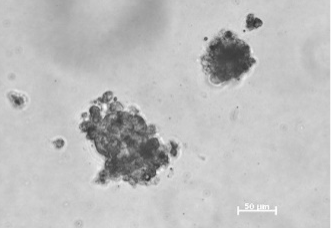

Supplement: Supplementary file 2 [file DataSheet_2.zip › Image 4.TIF]

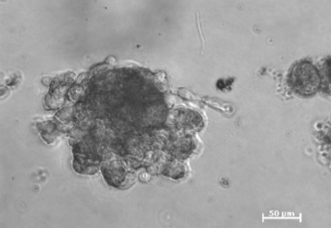

Supplement: Supplementary file 2 [file DataSheet_2.zip › Image 5.TIF]

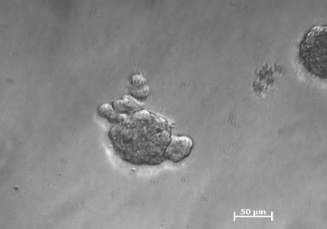

Supplement: Supplementary file 2 [file DataSheet_2.zip › Image 6.TIF]

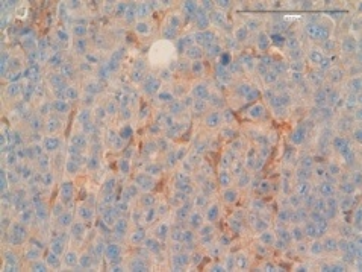

Supplement: Supplementary file 2 [file DataSheet_2.zip › Image 7.TIF]

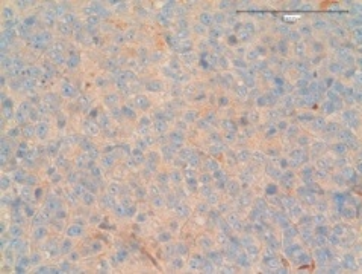

Supplement: Supplementary file 2 [file DataSheet_2.zip › Image 8.TIF]

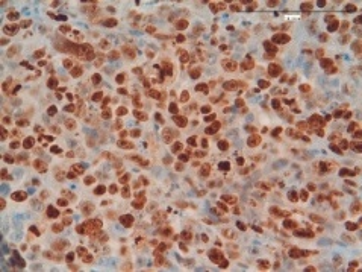

Supplement: Supplementary file 2 [file DataSheet_2.zip › Image 9.TIF]
